# A growing concern for meaning: Exploring the links between ego development and eudaimonia

**DOI:** 10.3389/fpsyg.2023.958721

**Published:** 2023-03-22

**Authors:** Evgeny N. Osin, Elena Yu. Voevodina, Vasily Yu. Kostenko

**Affiliations:** ^1^International Laboratory of Positive Psychology of Personality and Motivation, HSE University, Moscow, Russia; ^2^Laboratory LINP2-AAPS, University of Paris Nanterre, Nanterre, France

**Keywords:** ego development, eudaimonic orientations, implicit theories of meaning, lay theories, personal growth

## Abstract

**Introduction:**

Eudaimonia, in contrast to hedonia, is theorized to be a more complex type of positive functioning that involves personal growth and is guided by the pursuit of meaning. However, the existing evidence linking eudaimonia to personality development is rather scarce. To fill this gap, we aimed to explore whether ego development is related to eudaimonic well-being and eudaimonic orientations, most notably, the concern for meaning: we explored both the quantitative differences in the presence of meaning and the search for it, as well as qualitative differences in lay theories of meaning.

**Methods:**

Russian-speaking volunteers recruited online (*N* = 364, aged 18 to 85, 63% female) completed measures of ego development (Washington University Sentence Completion Test), meaning in life (Meaning in Life Questionnaire), lay theories of meaning (and original 20-item measure), hedonic and eudaimonic motives for activities (HEMA), and well-being (Mental Health Continuum—Short Form).

**Results:**

Ego development emerged as a weak, but significant positive predictor of well-being and this effect was fully mediated by the presence of meaning and eudaimonic motives. Latent profile analysis of the items tapping into lay theories of meaning revealed four distinct individual approaches to meaning that mainly differed in the subjective importance and salience of meaning. Participants with stronger concern for meaning revealed higher scores on ego development, both presence and search for meaning, eudaimonic motives, and well-being.

**Discussion:**

The results add to the evidence concerning the links between ego development and well-being and are in line with the theoretical view of eudaimonia as a process of growth guided by personal concern for meaning. The findings suggest that eudaimonia might be more easily attained by individuals at higher stages of personal development.

## Introduction

### Hedonia and eudaimonia

The research into eudaimonia and hedonia began with the question of whether they are different types of wellbeing or different pathways people use to seek wellbeing (Ryan and Deci, 2001; Kashdan et al., 2008). Recent theories and empirical findings suggest that eudaimonia and hedonia can be conceptualized as two distinct processes of positive functioning: they are both positively related to trait wellbeing indicators but are associated with different activities, motivational orientations, and emotional states (Huta, 2016; Vittersø, 2016, 2018).

Despite the rapidly growing popularity of this research field, consensus concerning an exact definition of eudaimonia is yet to be reached. Huta and Waterman (2014) carried out a systematic review of 11 existing models of eudaimonia and discovered two elements universally present in all of its definitions: growth (self-realization, self-actualization, development of potentials, full functioning, maturity) and meaning (purpose, long-term perspective, caring about and contributing to the broader context). To achieve conceptual clarity, they propose to distinguish eudaimonic orientations and behaviors (i.e., ways of living or a good life) from eudaimonic experiences and functioning (i.e., forms of wellbeing) (Huta and Waterman, 2014; Huta, 2016).

Our approach to eudaimonia and hedonia is informed by two theoretical contexts that offer symmetrical definitions of the two constructs. According to the Functional Wellbeing Approach (Vittersø, 2016, 2018), hedonic wellbeing is a subjective experience of pleasure or satisfaction that reflects homeostatic stability achieved by satisfying one's needs. Eudaimonic wellbeing, in turn, comprises experiences such as interest, engagement, curiosity, and awe, and reflects change or growth process associated with overcoming challenges. According to Vittersø (2018), the trait element of eudaimonic wellbeing is personal growth (Vittersø and Straume, 2017), which needs to be defined using both subjective and objective criteria and cannot be fully captured by self-reports. Another model distinguishing eudaimonia and hedonia at the level of trait-like motivational orientations was proposed by Huta (2016). She differentiates hedonic motives, which comprise striving for pleasure and comfort, avoidance of distress and negative emotions, from eudaimonic motives comprising the pursuit of personal growth, meaning, authenticity, and excellence.

These two models appear to complement each other, providing a clear distinction between eudaimonia and hedonia at the trait and state levels. Eudaimonia emerges from these models as a more complex and resource-demanding type of positive functioning based on effortful action in pursuit of meaning or virtue, in contrast to hedonia, which is relatively effortless and more readily available in many life situations. Therefore, eudaimonia might be based on more complex cognitive processing and more mature personality structures, processes, and resources developed throughout the lifespan. In the present research, we aimed to explore the relationships of eudaimonic orientations and eudaimonic wellbeing with personal growth and development.

### Developmental basis of eudaimonia

The idea that eudaimonia (understood in terms of orientations or wellbeing outcomes) is related to maturity has been proposed by several authors (Bauer, 2016; Law and Staudinger, 2016; Ryff, 2016). Bauer and McAdams (2010) and Bauer (2016) defined eudaimonic growth as a process of a parallel increase in subjective wellbeing (SWB) and psychosocial maturity understood as complexity and integration in the ways of thinking about one's life. More recently, Bauer et al. (2015) have differentiated two interrelated motivational facets of eudaimonic growth, reflective (aiming to develop new perspectives on oneself, others, and life), which is more strongly associated with indicators of psychosocial maturity, and experiential (aiming to cultivate meaningful activities and relationships), more strongly related to wellbeing outcomes.

However, empirical support for the link between eudaimonia and maturity is still scarce, as the latter is rather difficult to measure. According to Bauer et al. (2015), maturity is an umbrella term that covers a range of constructs describing distinct yet related aspects of cognitive and personality development (such as wisdom, self-actualization, moral reasoning, and ego development, among others). The challenges of measuring these notions with self-report instruments have been discussed extensively (Nisbett and Wilson, 1977; Kunzmann, 2019): King (2011) notes that scholars tend to blur the lines between the conscious feelings of personal growth and the more objective personality development that do not necessarily have much to do with each other. In operationalizing maturity, we relied on Loevinger's (1976) Ego Development (ED) theory which unifies cognitive and personality development and proposes an elaborate performance-based empirical operationalization of maturity.

According to Loevinger (1976), ego is a holistic construct representing the structural unity of personality organization that unifies both the integrative processes a person uses to deal with life experiences and the frame of reference she/he subjectively imposes on these experiences to create meaning. The ED theory describes a sequence of nine developmental stages or levels (labeled E1–E9) that reflect a progressive reorganization of the self and are defined by characteristic features of impulse control and moral character, cognitive style, interpersonal style, and conscious preoccupations.

The first, symbiotic, stage of ego formation (E1) is pre-conscious and is not accessible for assessment. At the earlier, Impulsive (E2) and Self-Protective (E3) stages, individuals are predominantly self-focused and are preoccupied with bodily feelings and with controlling their environments in order to obtain gratification. At the Conformist (E4) stage, individuals identify with the group, rely on rules and conventions in shaping their behavior, and are preoccupied with appearance and belonging. The Self-Aware (E5) stage brings in a limited awareness of possible exceptions, as well as of one's individuality and inner life. It is followed by the Conscientious (E6) stage when individuals start to rely on self-evaluated standards and ideals and to think beyond their personal concerns. The Individualistic (E7) stage brings in a sense of one's personality as a whole, as well as recognition of that of others, and a preoccupation with finding a balance between one's needs, wishes, and obligations. At the Autonomous (E8) stage, individuals become aware of the complexity of social interactions and are able to acknowledge and accept unresolvable conflicts, develop a broader scope of concern and focus on the search for self-fulfillment. The Integrated (E9) stage corresponds to Maslow's view of a self-actualizing person and involves the search for identity. To assess the ED level, Loevinger developed the Washington University Sentence Completion Test (WUSCT; Hy and Loevinger, 1996), a projective measure with extensive evidence of reliability and validity (Gilmore and Durkin, 2001).

Within the field of eudaimonia, ED has been proposed as a measure of psychosocial maturity or practical wisdom that reflects eudaimonic functioning (Bauer, 2016; Huta, 2016). Indeed, the descriptions of stages in ED theory indicate that the behaviors and motivations described within various models of eudaimonia (such as the pursuit of excellence, authenticity, autonomy, personal growth, autotelic engagement, and acceptance of reality) (Huta and Waterman, 2014) become increasingly salient throughout the progression from conventional to post-conventional ED stages (E5–E9). Nevertheless, despite the strong theoretical affinity between these two research fields, the empirical evidence concerning the links between eudaimonia and maturity is still limited. We aimed to contribute to this body of evidence by investigating the associations of ego development with the eudaimonic motivational orientations (most notably, concern for meaning) and with the wellbeing outcomes of eudaimonic functioning.

### Ego development and the growing concern for meaning

Within Loevinger's approach, “ego” is understood as the underlying principle in personality organization that develops and generates coherent meaning: “the search for coherent meanings in experience is the essence of ego or ego functioning, rather than just one among many ego functions” (Hy and Loevinger, 1996). The levels of ED reflect distinct forms of meaning-making, templates, or frameworks that individuals continuously apply to their experiences. As the ED level increases, individuals gradually progress from a rigid and simplified understanding of the world (e.g., dichotomous “good vs. bad” evaluations) to increased complexity and integration of thinking about oneself and others. Instead of accepting culturally programmed meanings, they become increasingly aware of themselves as meaning-makers and conscious of their own meaning-making activity and its limits (Loevinger, 1976; Hauser, 1993; Cook-Greuter, 1999).

Based on ED theory, one can expect that at higher ED stages the theme of meaning becomes increasingly salient with the search for meaning emerging as a conscious concern. Many prominent theories of meaning emphasize its universal aspects: for instance, Frankl (1969) believed that the search for meaning is a basic and primary motivation, therefore, the question of meaning is inevitably faced by every human being. Many other theorists also viewed the need for meaning, understood either as a cosmic, self-transcendent purpose or frame of orientation, or a more mundane, existential meaning related to the value behind one's everyday actions, as a universal human need (Baumeister, 1991; Längle, 2005; Fromm, 2011). However, some authors suggested that the need for meaning or purpose only emerges at higher levels of personality development (Jung, 1954; Maslow, 1971). The latter stance seems to be more in line with recent empirical findings revealing “existential indifference”: some individuals report an absence of meaning and no desire to seek it (Schnell, 2010). Still other existentialist and psychodynamic approaches to meaning suggest that it emerges with time as a general direction of actions taken and decisions made by an individual and may only be consciously recognized in retrospect (Adler, 1958; Maddi, 2012).

This diversity of views leads to the question of whether ego development involves facing the problem of meaning as a personal and conscious problem. This question is further complicated by the very ambiguity of the concept of meaning, which may manifest itself in emotional experiences, such as the meaningfulness or significance of one's actions or life as a whole, in cognitive constructions, such as views regarding one's life purpose or direction, in motivational processes, such as actual or possible goals, and in one's behavior, as an emergent direction of one's actions and daily activities (Leontiev, 2013; Martela and Steger, 2016). The common self-report instruments measuring meaning often focus on some of these aspects or rely on a subjective understanding of the term “meaning” (Brandstätter et al., 2012), adding further to the confusion.

In the present study, we sought to explore whether individuals at different levels of ED would exhibit differences in their understanding of the concept of meaning and in their views regarding its importance. To address the individual diversity of views regarding meaning, we complemented quantitative assessment of the presence and search for meaning with a novel instrument exploring the qualitative differences in lay (implicit) theories of meaning using the person-oriented approach (Bergman and Magnusson, 1997). We used a set of items tapping into the nature of meaning, its origins and availability, as well as its necessity for human life and its personal salience, in order to uncover holistic distinct patterns (or common types) of view regarding meaning—implicit theories of meaning.

### Ego development and wellbeing

Given that eudaimonia comprises ways of behaving and forms of wellbeing (Huta, 2016), we approach the final question, whether ego development brings about higher wellbeing. This question has been a matter of considerable debate. According to Loevinger (1968), ego development is “conceptually distinct” from the health-illness dimension, and it is only at the low end of the ED continuum that a “direct relation between ego development and mental health, adjustment or pathology is found” (Loevinger, 1968, p. 170). She suggested that while lower ED stages tend to be associated with maladjustment, the kinds or symptoms of psychopathology tend to differ at different ED stages; correspondingly, the criteria of mental health have to differ as well with the conventional criteria of mental health only applying to individuals at higher ED stages (Loevinger, 1968, 1976).

Empirical data are generally consistent with these ideas: numerous studies show that higher ED levels are associated with greater internalization of distress and readiness for psychotherapy (Noam, 1998; Duffy et al., 2017), however, the associations of ED with wellbeing measures found in different studies are very modest in magnitude, rarely attaining *r* = 0.20, or even non-existent (Noam, 1998; King and Hicks, 2007; Bauer and McAdams, 2010; Bauger et al., 2021). Until meta-analytic studies are conducted, it is hardly possible to conclude whether ego development is completely independent of wellbeing and psychological adjustment (Noam, 1998; King and Hicks, 2007), but, at least, their associations appear too weak to be routinely detected, given typical sample sizes in the field.

If higher complexity and maturity are supposed to facilitate self-regulation, coping, and adjustment, why are the links between ED and wellbeing so weak? One possible explanation is that pronounced changes in wellbeing may only pertain to the highest ED stages (Bauer, 2011; Bauer et al., 2011) that are rarely found in the general population. In addition, higher complexity associated with these stages brings about greater awareness of conflict and the desire for self-actualization might result in more difficulty fitting in with the social system (Maslow, 1971; Pals and John, 1998): the processes of growth and adjustment are different and may not always lead in the same direction (Law and Staudinger, 2016).

Another explanation is that the changes in wellbeing associated with maturity are more qualitative than quantitative (Fossas, 2019) and are related to the more objective (i.e., activity) aspects of positive functioning than to its subjective perception. Certainly, subjective wellbeing measures (Diener, 2009) have numerous advantages, one of them being content-free (Sheldon, 2018), in line with Loevinger's (1968) early idea that the definition of mental health should not be broadened. However, they tend to contain a mixture of phenomenological indicators of hedonic and eudaimonic states (Vittersø, 2016) and also fail to address complex emotions, such as awe, elevation, or fulfillment (Huta, 2013). Extending self-report measures to address the diverse facets of positive functioning may not solve the problem either: similar subjective evaluations of autonomy, competence, relationships, etc. at different ED levels may conceal the differences in the complexity of their objective manifestations and/or in the subjective criteria used to evaluate them. However, the issue of measurement of eudaimonic wellbeing is far from being resolved at present.

Given the scarcity of existing evidence concerning the link between wellbeing and ED, we sought to re-examine it in a new cultural setting using measures of eudaimonic wellbeing and eudaimonic orientations.

### Study aims

We focused on two principal aims:

Firstly, we aimed to explore the associations of ED with wellbeing, meaning in life, and hedonic and eudaimonic motives. Building on Bauer (2011), we hypothesized that ED should be positively associated with eudaimonic wellbeing and that their shared variance should be fully mediated by eudaimonic orientations (meaning and eudaimonic motives) that become more prominent as one attains maturity.

Secondly, we aimed to explore the associations of ED with lay theories of meaning in life and views regarding its importance. Based on existing theory (Loevinger, 1976; Cook-Greuter, 1999), we expected that higher ED levels would be associated with a greater diversity of views regarding the nature of meaning and higher importance ascribed to meaning.

## Methods

### Participants and procedure

The study used a cross-sectional design in a sample of 364 Russian-speaking adults, 133 male and 231 female, between 18 and 85 years old (*M* = 30.1, *SD* = 11.5). Most participants (88.5%) had higher education or were current students (33.5% with a Bachelor's degree, 40.7% with a Master's degree, and 14.3% with an advanced degree). Initially, we had aimed for a minimum *N* = 193 allowing us to detect a typical effect size (*r* = 0.20) with 80% power. However, given the high response rate and the possibility of weaker effects, we opted to collect as large a sample as possible, achieving 80% power for weaker effects (*r* = 0.15, according to sensitivity analysis) with our final sample size.

The research was conducted in accordance with the Declaration of Helsinki. Participants were anonymous volunteers invited *via* social networks and online communities to take part in an online survey of views on life meaning and personality traits. After providing their informed consent and completing the questionnaires, they could opt-in to receive an update on the study results; no remuneration of any kind was provided. The study protocol was approved by the HSE University Psychology Research Ethics Committee.

### Instruments

#### Washington University Sentence Completion Test

The WUSCT (Loevinger, 1985; Hy and Loevinger, 1996; Russian validation: Leontiev and Kostenko, in preparation) includes 36 open-ended sentences (e.g., “When people are helpless…”) the respondents are asked to complete. During scoring, each response is assigned an ED level ranging from E2 (Impulsive) to E9 (Integrated), and the whole protocol is assigned an “impressionist” rating. The WUSCT has high reliability and extensive evidence of construct, predictive, and discriminant validity (reviewed in Gilmore and Durkin, 2001).

In the present study, we used a short form of the WUSCT including the first 18 items (Holt, 1980; Loevinger, 1985). First, each protocol was scored by an experienced rater with 4 weeks of training. Next, the scoring was checked by another rater with several years of experience in coding the WUSCT. Next, we used the item sum score approach recommended by Hy and Loevinger for the 18-item version to generate the total protocol rating (TPR) for each individual (essentially, a sum score of the items). Finally, we used the sum rule (Loevinger, 1998) to classify the protocols into discrete ED stages. Spearman correlation of impressionist rating with the TPR and the ED score based on the sum rule was ρ = 0.89 and 0.88, respectively (*p* < 0.001).

#### Hedonic and Eudaimonic Motives for Activities-Revised

Hedonic and Eudaimonic Motives for Activities-Revised (HEMA-R) (Huta, 2015) consists of 11 items rated on a 5-point scale. The instructions for the trait version ask to rate the degree to which the participants approach their activities with each of the intentions listed. Six items assess hedonic motives reflecting the pursuit of pleasure and relaxation (sample item: “Seeking to take it easy”) and five items assess eudaimonic motives reflecting the pursuit of authenticity, excellence, and growth (sample item: “Seeking to do what you believe in”). The internal consistency coefficients for all the study measures are given in Table 1.

**Table 1:** Descriptive statistics for the individuals at different ego development stages, *M* (SD).

	**Pre-conventional E2–E3 (*N* = 28)**	**Conformist E4 (*N* = 57)**	**Self-aware E5 (*N* = 138)**	**Conscientious E6 (*N* = 98)**	**Post-conventional E7–E9 (*N* = 41)**
Hedonic motives	5.25 (0.95)	4.93 (1.12)	4.92 (0.93)	5.14 (0.93)	5.08 (0.83)
Eudaimonic motives	5.30 (1.03)	5.38 (0.92)	5.40 (0.90)^*^	5.73 (0.74)^***^	5.85 (0.68)^**^
Presence of meaning	4.22 (1.40)	4.08 (1.79)	4.35 (1.49)	4.47 (1.51)	4.82 (1.45)
Search for meaning	4.41 (1.29)	3.88 (1.50)	4.38 (1.33)^*^	4.42 (1.50)	4.40 (1.34)
Emotional wellbeing	3.50 (1.37)	3.56 (1.36)	3.71 (1.29)	3.87 (1.19)^*^	3.98 (1.09)
Social wellbeing	2.79 (1.03)	2.84 (1.05)	2.87 (0.99)	2.92 (1.01)	3.27 (1.13)^*^
Psychological wellbeing	3.51 (1.17)	3.49 (1.05)	3.61 (1.12)	3.66 (0.98)	3.87 (1.04)
MHC total score	3.25 (1.07)	3.27 (0.99)	3.37 (0.99)	3.44 (0.87)	3.68 (0.96)^*^

The ED stages were determined based on sum rule (Loevinger, 1998). Significance of the difference between the combined score of individuals at this and the subsequent stages from that of the individuals at prior stages combined (Welch's t-test): ^***^p < 0.001, ^**^p < 0.01, ^*^p < 0.05.

#### Mental Health Continuum—Short Form

Mental Health Continuum—Short Form (MHC-SF) (Keyes et al., 2011; Russian validation: Zemojtel-Piotrowska et al., 2018) includes 14 items reflecting various experiences of wellbeing whose frequency over the past month the participants are asked to rate using a 6-point scale. The items tap into emotional wellbeing (happiness, interest, and satisfaction), social wellbeing (social contribution, integration, growth, acceptance, and coherence), and psychological wellbeing (self-acceptance, environmental mastery, positive relations, personal growth, autonomy, and purpose in life).

#### Meaning in Life Questionnaire

Meaning in Life Questionnaire (MLQ) (Steger et al., 2006; Russian version: Osin et al., 2014) is a brief measure of meaning with 10 items rated on a 5-point scale. It includes two five-item subscales, Presence of Meaning (sample item: “My life has a clear sense of purpose”) and Search for Meaning (sample item: “I am seeking a purpose or mission for my life”).

#### Implicit Theories of Life Meaning

Implicit Theories of Life Meaning (ITLM) (Osin et al., 2014) is a semantic-differential-type measure that attempts to capture the diversity of individual views concerning the nature, origins, and necessity of meaning in life. It includes 20 items (six unipolar and 14 bipolar ones) rated on a 5-point scale and reflecting the diversity of existing theoretical positions on the meaning of life (the complete list of items is given in Table 2).

**Table 2:** Descriptive statistics and correlations for the study measures.

**Variable**	**1**	**2**	**3**	**4**	**5**	**6**	**7**	**8**	**9**	**10**	**11**	**12**
1. Ego development (TPR)												
2. Ego development (Sum rule)	0.96^**^											
3. Hedonic motives	0.01	0.07										
4. Eudaimonic motives	0.21^**^	0.19^**^	0.29^**^									
5. Presence of meaning	0.13^*^	0.11^*^	0.07	0.52^**^								
6. Search for meaning	0.11^*^	0.09	−0.07	0.08	−0.12^*^							
7. Emotional wellbeing	0.11^*^	0.11^*^	0.16^**^	0.43^**^	0.53^**^	−0.05						
8. Social wellbeing	0.10	0.09	0.09	0.34^**^	0.48^**^	0.02	0.60^**^					
9. Psychological wellbeing	0.09	0.09	0.11^*^	0.47^**^	0.59^**^	−0.05	0.73^**^	0.65^**^				
10. MHC-SF total wellbeing	0.11^*^	0.12^*^	0.13^*^	0.47^**^	0.61^**^	−0.03	0.85^**^	0.86^**^	0.92^**^			
11. Gender (0 = male, 1 = female)	0.13^*^	0.08	−0.05	0.03	0.05	0.18^**^	0.18^**^	0.18^**^	0.09	0.16^**^		
12. Age	−0.01	−0.03	−0.21^**^	0.10	0.23^**^	−0.08	0.21^**^	0.20^**^	0.25^**^	0.25^**^	0.10^*^	
13. Education	−0.01	−0.02	−0.12^*^	0.10	0.16^**^	−0.03	0.14^**^	0.11^*^	0.14^**^	0.15^**^	0.06	0.52^**^
α	0.89	n/a	0.74	0.69	0.90	0.86	0.82	0.70	0.78	0.89		
M	4.92	5.20	5.02	5.53	4.41	4.33	3.75	2.92	3.63	3.40		
SD	0.55	1.15	0.95	0.87	1.53	1.39	1.26	1.03	1.07	0.97		

* *p* < 0.05.

** *p* < 0.01.

Pairwise N ranges from 345 to 362. Spearman correlations are given for categorical variables (ego development based on sum rule and education).

n/a – not available: the score based on sum rule is a discretization of the Total Protocol Rating (TPR) score.

Given that the views concerning life meaning may have complex and individually-specific structure, the ITLM items are not grouped into scales, but, rather, are supposed to be analyzed using a person-oriented approach methodology. A previous study (Osin et al., 2014) using hierarchical cluster analysis has found four groups of individuals with distinct approaches to meaning (viewing meaning as a goal or direction of life, as a subjective experience, as something vague but potentially useful, and as an absurd question). The groups also showed predictable differences in the scores on explicit measures of life meaning and personality resources. In the present study, we used a more robust Latent Profile Analysis (LPA) methodology to uncover the structure of views regarding life meaning.

### Data analysis

Nineteen participants failed to complete one or more measures or provided invalid responses (following Curran, 2016, we screened out responses from participants who provided the same answer to a series of 10 or more questions). The number of missing responses ranged from 2 to 14 per measure (two for WUSCT and MHC-SF, seven for MLQ, and 14 for HEMA). Little's MCAR test was not significant, indicating that the data were missing completely at random. As a result, we opted to use weighted least squares estimation in Mplus for the latent variable models and to report pairwise *N* for the analyses based on observed variables.

First, we explored pairwise associations between ED stages and the other study variables. The results based on the three versions of WUSCT scoring (Total Protocol Rating, discrete score based on sum rule, and impressionist scoring of the whole protocol) were convergent. We calculated correlations of the ED scores with the other variables, and compared individuals at different ED stages using one-way ANOVA.

Next, we tested the mediation models using SEM in Mplus 8.4 with the WLSMV estimator for categorical items. We used conventional criteria to evaluate fit indices (Hu and Bentler, 1999), interpreting them in combination (Brown, 2015). First, we tested a theoretical measurement model for each questionnaire using ICM-CFA and ESEM (the parameters of measurement models are given in Supporting Information). Next, we proceeded by testing structural models where the association of ED with a latent wellbeing factor defined by the three MHC subscale scores was mediated by HEMA and MLQ scales. In each case, we tested a partial mediation model with correlated mediators and applied the Wald test to find out whether constraining the direct path from ED to MHC to zero would adversely affect the model fit.

To investigate the existence of distinct lay theories of life meaning, we applied latent profile analysis in Mplus to the 20 ITLM items. The variables were modeled as ordered categorical. We used 10,000 random starts with 50 initial stage iterations and 2,500 final stage optimizations. In choosing a model, we relied on entropy, information criteria, likelihood ratio tests (Asparouhov and Muthén, 2012), and theoretical interpretability in combination. We compared models with two–five latent classes. With five classes, the model became unstable and showed poor convergence (45% of initial-stage solutions failed to converge). The model fit statistics (given in Supporting Information) generally favored models with a larger number of classes, except for BIC and VLMRT, which suggested two and three classes, respectively. Based on the combination of statistical criteria, model convergence, and theoretical considerations, we opted for the model with 4 latent classes.

In order to interpret the class profiles, we compared the groups based on the most likely class membership on the ITLM items using Kruskal–Wallis ANOVA. To establish significant pairwise differences between the groups, we used the Conover-Iman *post-hoc* test procedure (Conover and Iman, 1979) with Benjamini-Hochberg correction (Benjamini and Hochberg, 1995) for multiple comparisons implemented in the R package PMCMR. Finally, we compared the groups on the other variables using ANOVA with the Tukey *post-hoc* test.

## Results

### Ego development and eudaimonia

In terms of the distribution of individuals across the ED stages, the sample was consistent with existing findings, revealing a prevalence of conventional ego stages (see Table 1). The modal category was E5 “Self-aware” (38.1%), followed by E6 “Conscientious” (27.1%) and E4 “Conformist” (15.7%). The proportions of individuals scoring at post-conventional (E7–E9) and pre-conventional (E2-E3) stages were fairly low (11.3 and 7.7%, respectively).

The distribution of mean scores across the groups of individuals with different ED levels is given in Table 1. Only the difference in eudaimonic motives was significant across the five groups (*F*_(4, 343)_ = 4.29, *p* = 0.002, η^2^ = 0.048) with Tukey *post-hoc* test indicating higher scores (*p* < 0.05) in the groups E6 “Conscientious” and E7–E9, compared to E5 “Self-Aware.”

The correlations (presented in Table 2) revealed weak positive associations of ED with eudaimonic motives, presence of meaning, search for meaning, emotional wellbeing, and the total MHC-SF score. Eudaimonic motives were the strongest correlate of ED. Predictably, eudaimonic motives and the presence of meaning were correlated with each other and with wellbeing. Search for meaning was only associated with ED and, inversely, with the presence of meaning. Hedonic motives only showed weak positive associations with emotional and psychological wellbeing.

Demographic variables (gender, age, and education) revealed weak associations with the study variables. Wellbeing tended to be higher in female participants, older adults, and those with higher education. The presence of meaning was only positively associated with age and education, whereas female participants showed higher scores on the search for meaning scale. Hedonic motives were weaker in older participants and those with higher education. ED was only marginally related to gender with higher scores in female participants.

#### Mediation models

The first model, where the association between ED and wellbeing was fully mediated by hedonic and eudaimonic motives, fit the data well [χ^2^ = 749.10, df = 457, *p* < 0.001; CFI = 0.940; RMSEA = 0.042, 90% *CI* (0.036, 0.047); SRMR = 0.053]. Hedonic motives were not significantly associated with ED [*a*_1_ = 0.076, 95% *CI* (−0.025; 0.177), *p* = 0.139] and wellbeing [*b*_1_ = −0.089, 95% *CI* (−0.223; 0.046), *p* = 0.196]. Eudaimonic motives, in turn, were predicted by ED [*a*_2_ = 0.257, 95% *CI* (0.150; 0.363), *p* < 0.001] and predicted wellbeing [*b*_2_ = 0.647, 95% *CI* (0.496; 0.799), *p* < 0.001]. The specific indirect effect of ED on wellbeing mediated by eudaimonic motives was significant [*a*_2_*b*_2_ = 0.166, 95% *CI* (0.083; 0.250), *p* < 0.001], and the Wald test supported the full mediation hypothesis [χ^2^ (1) = 0.42, *p* = 0.52].

The second model, where the association of ED and wellbeing was mediated by the presence of meaning and the search for meaning, also showed a good fit to the data [χ^2^ = 779.78, df = 428, *p* < 0.001; CFI = 0.958; RMSEA = 0.048, 90% *CI* (0.042, 0.053); SRMR = 0.052]. ED was a significant predictor of both presence of meaning [*a*_1_ = 0.147, 95% *CI* (0.040; 0.253), *p* = 0.007] and search for meaning [*a*_2_ = 0.119, 95% *CI* (0.016; 0.222), *p* = 0.024]. Wellbeing, however, was only predicted by the presence of meaning [*b*_1_ = 0.680 (0.606; 0.755), *p* < 0.001], but not by the search for meaning [*b*_2_ = 0.071 (−0.016; 0.158), *p* = 0.111]. The specific indirect effect of ED on wellbeing mediated by the presence of meaning was significant [*a*_1_*b*_1_ = 0.100, 95% *CI* (0.026; 0.174), *p* = 0.010] and the Wald test, again, supported full mediation [χ^2^ (1) = 0.09, *p* = 0.77].

**Figure 1:**
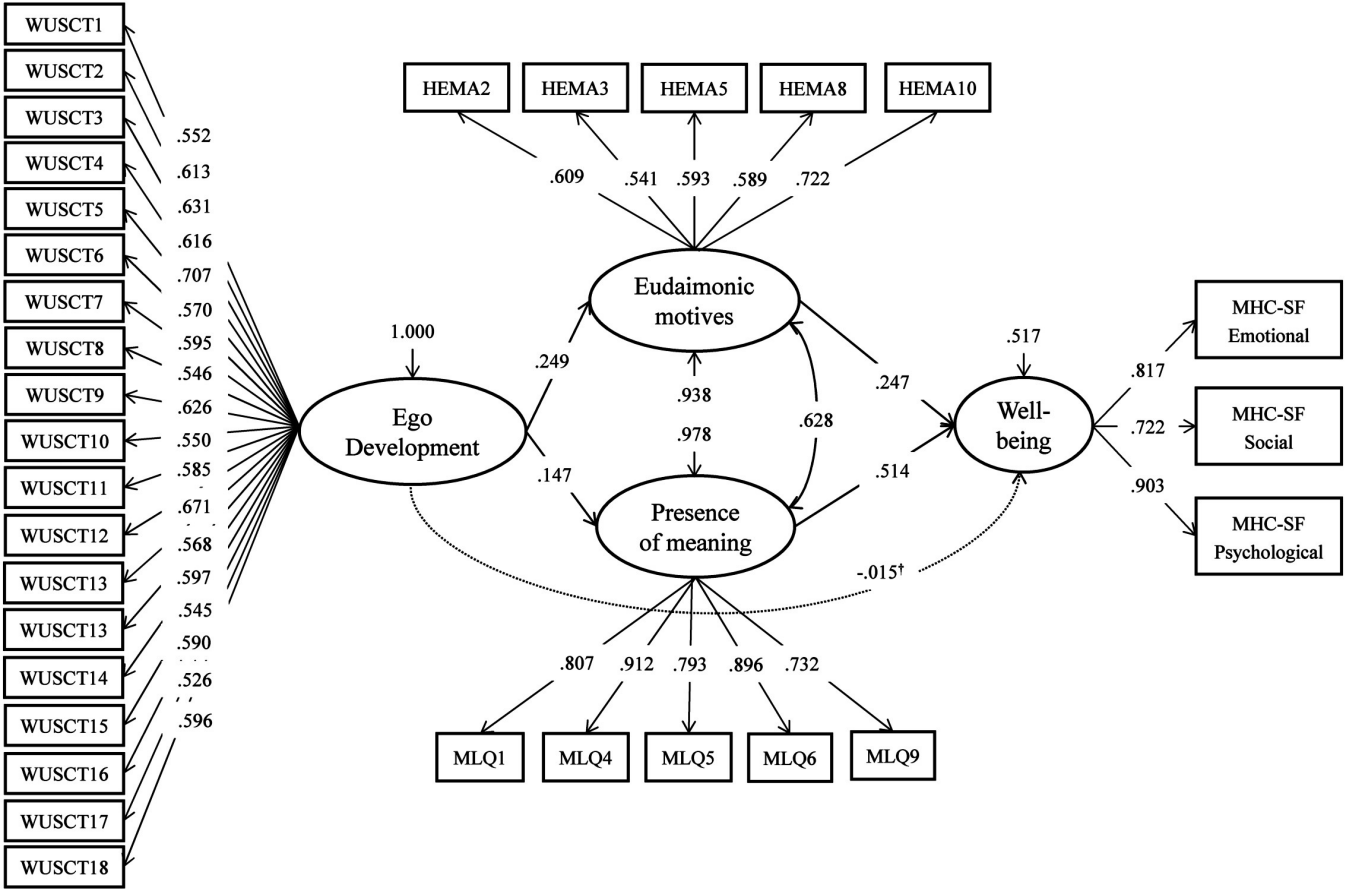
Parameters of the parallel mediation model. χ^2^ = 627.22, df = 428, *p* < 0.001; CFI = 0.973; RMSEA = 0.036, 90% *CI* (0.030; 0.042); SRMR = 0.048. Standardized coefficients are shown; all the parameters are significant at *p* < 0.05, except for those marked^†^.

Finally, we explored whether eudaimonic motives and the presence of meaning would independently contribute to explaining the shared variance of ED and wellbeing in a single model. The fit indices and parameters of this parallel mediation model are given in Figure 1. Predictably, the Wald test supported full mediation [χ^2^ (1) = 0.10, *p* = 0.76]. Both specific indirect effects of ED on wellbeing mediated by the presence of meaning [*a*_1_*b*_1_ = 0.076, 95% *CI* (0.017; 0.134), *p* = 0.011] and by eudaimonic motives [*a*_2_*b*_2_ = 0.061, 95% *CI* (0.014; 0.109), *p* = 0.011] were significant and comparable in magnitude. The results were substantially the same when each of the three MHC scales was modeled individually as a latent dependent variable (full mediation with both indirect effects significant and in the 0.060–0.080 range).

Following the Reviewers' suggestions, we tested two additional models. In the first model, we entered demographic variables as covariates of all four latent factors to control for their effects. The model fit the data well [χ^2^ = 718.09, df = 509, *p* < 0.001; CFI = 0.973; RMSEA = 0.034, 90% *CI* (0.028, 0.039); SRMR = 0.055]. Out of demographic variables, only gender emerged as a significant positive predictor of ED (β = 0.146, *p* = 0.006) and wellbeing (β = 0.147, *p* = 0.001) and only age emerged as a predictor of the presence of meaning (β = 0.204, *p* < 0.001). Nevertheless, the estimates of direct and indirect effects did not change substantially: again, both specific indirect effects of ED on wellbeing mediated by the presence of meaning [*a*_1_*b*_1_ = 0.069, 95% *CI* (0.017; 0.122), *p* = 0.010] and by eudaimonic motives [*a*_2_*b*_2_ = 0.065, 95% *CI* (0.017; 0.113), *p* = 0.008] were significant and comparable in magnitude.

**Figure 2:**
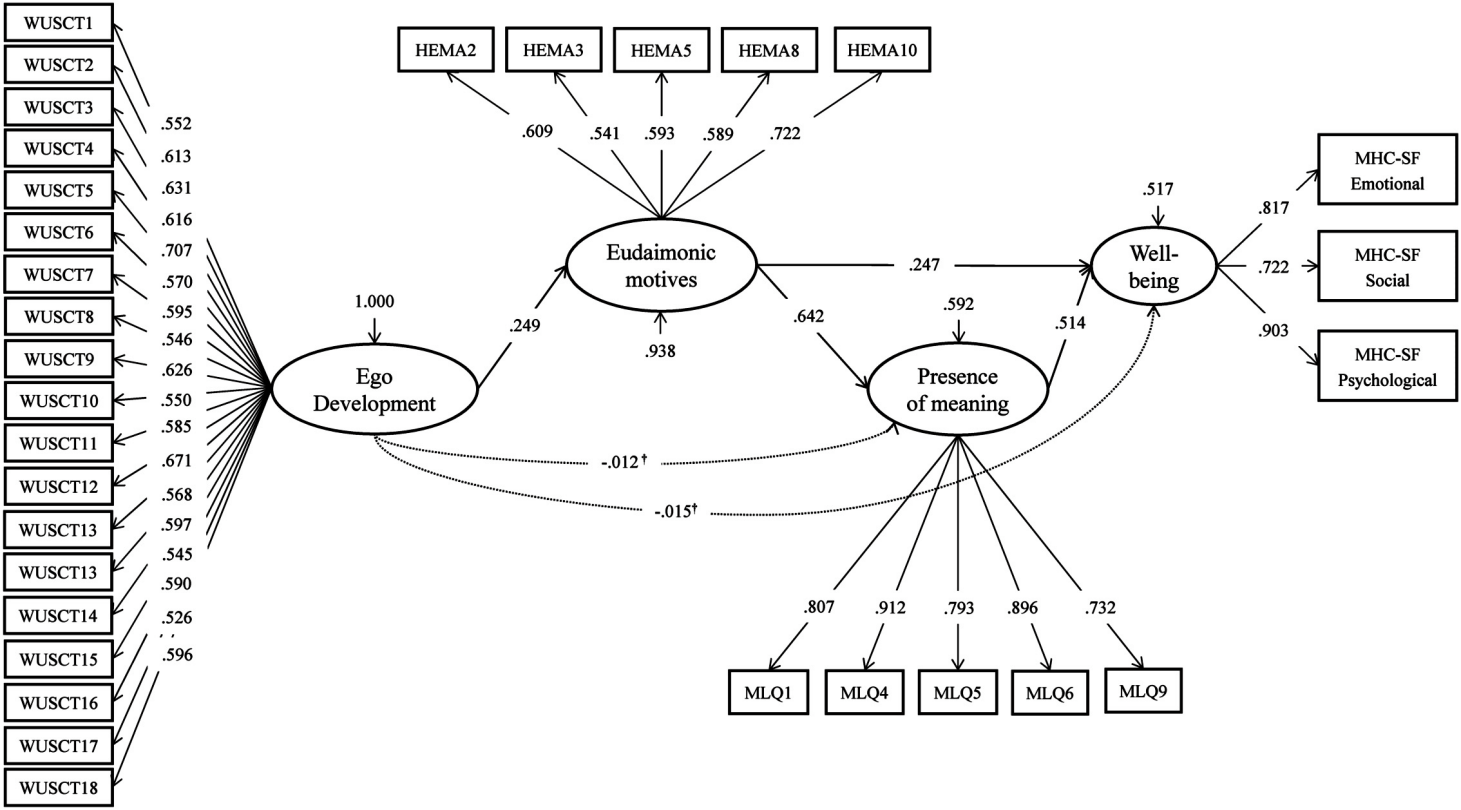
Parameters of the serial mediation model. χ^2^ = 627.22, df = 428, *p* < 0.001; CFI = 0.973; RMSEA = 0.036, 90% *CI* (0.030; 0.042); SRMR = 0.048. Standardized coefficients are shown; all the parameters are significant at *p* < 0.05, except for those marked^†^.

We also tested an alternative model with serial partial mediation, where ED predicted eudaimonic motives, which, in turn, predicted the presence of meaning, and the latter predicted wellbeing. This serial mediation model (see Figure 2) was mathematically equivalent, whose fit indices are presented in Figure 1. The estimate of the specific indirect effect reflecting serial mediation of the effect of ED on wellbeing by eudaimonic motives and the presence of meaning was statistically significant [a_1_b_1_c_1_ = 0.082, 95% CI (0.039; 0.125), *p* < 0.001]. However, the Wald test rejected the full mediation hypothesis [χ^2^ (3) = 9.95, *p* = 0.02] due to a significant direct effect of eudaimonic motives on wellbeing (*b*_2_ = 0.247, *p* < 0.001).

#### Ego development and lay theories of life meaning

Descriptive statistics and correlations with ED for the individual items tapping into lay theories of meaning are given in Table 3. Correlation analysis has revealed that individuals with higher ED levels are more likely to understand meaning as a rational idea or an emotional subjective experience (items 1 and 2). They are more likely to see meaning as a possibility that can be accomplished (item 10), a reality that is created and shaped by one's conscious choices, rather than a given that does not depend on the person(items 11 and 14). Finally, individuals at higher ED stages are more likely to see the question of meaning as a personally important and reasonable one, rather than useless or impossible to answer (items 6, 16, 17, 19).

**Table 3:** Descriptive statistics and Spearman correlations with ED (*N* = 362) for the ITLM items.

	***M* (SD)**	**Mean in group**				***E* ^2^**	**ρ_ED_**
		**1**	**2**	**3**	**4**		
**Life meaning is…**							
1. An idea, a conscious notion	3.59 (1.20)	3.60_ab_	3.22_a_	3.53_a_	3.96_b_	0.068^***^	0.13^*^
2. An experience, a feeling	3.60 (1.22)	3.59_a_	3.57_a_	3.69_a_	3.53_a_	0.002	0.14^**^
3. Presence of a goal in life	3.78 (1.15)	3.20_a_	3.47_a_	4.01_b_	3.95_b_	0.063^***^	−0.06
4. A general direction of one's life	3.76 (1.12)	3.50_a_	3.36_a_	4.01_b_	3.89_b_	0.063^***^	−0.02
5. An illusion, as life is absurd	2.40 (1.40)	3.55_a_	3.33_a_	1.85_b_	1.91_b_	0.234^***^	−0.02
6. A question useless to ponder on	2.15 (1.33)	3.63_a_	3.17_a_	1.74_b_	1.28_c_	0.432^***^	−0.14^**^
**Life meaning…**							
7. Is possible to understand … Is only possible to feel, experience	3.02 (0.98)	3.10_a_	3.55_b_	2.94_a_	2.66_a_	0.116^***^	−0.06
8. Every person has it … Few people have it	3.26 (1.36)	3.85_a_	3.56_ac_	3.01_b_	3.10_bc_	0.056^***^	−0.07
9. Is necessary for one's life … Is possible to live without	3.07 (1.27)	3.93_a_	3.69_a_	2.71_b_	2.68_b_	0.166^***^	0.04
10. Is a given that does not depend on one … Is a possibility one can accomplish	4.00 (1.02)	3.37_a_	3.95_ab_	3.90_b_	4.42_c_	0.100^***^	0.22^***^
11. One has to create it … One can discover or understand it	3.17 (1.06)	3.53_a_	2.82_b_	3.20_a_	3.28_a_	0.046^**^	−0.14^**^
12. Is specific to each individual …Is common, universal	2.07 (1.06)	2.28_ab_	1.94_ab_	2.19_a_	1.94_b_	0.022^*^	−0.09
13. Can only be present if found consciously … Can be present even if one never thought about it	3.34 (1.12)	3.33_a_	3.41_a_	3.36_a_	3.25_a_	0.001	0.01
14. Emerges as a result of one's deliberate choices … Exists regardless of one's choice	2.64 (1.02)	2.90_a_	2.70_a_	2.81_a_	2.29_b_	0.062^***^	−0.17^**^
**The question of meaning…**							
15. Is faced by every person … Is only faced by few people	2.76 (1.11)	3.12_a_	2.88_a_	2.59_a_	2.74_a_	0.021	0.03
16. Is a reasonable question … Is a pointless question	2.33 (1.09)	3.78_a_	2.93_b_	2.21_c_	1.44_d_	0.477^***^	−0.13^*^
17. Has a lot to do with me … Has nothing to do with me	1.91 (1.09)	2.93_a_	2.52_a_	1.74_b_	1.25_c_	0.301^***^	−0.20^***^
18. Is bothering me at present …Does not bother me at present	2.96 (1.38)	3.90_a_	3.22_b_	2.72_c_	2.66_c_	0.084^***^	−0.09
19. Can be answered … Is impossible to find an answer to	2.45 (1.14)	3.46_a_	3.29_a_	2.32_b_	1.56_c_	0.398^***^	−0.18^***^
20. Emerges when life goes well …Emerges when something goes wrong in one's life	3.37 (0.75)	3.60_a_	3.47_a_	3.28_a_	3.32_a_	0.026^*^	−0.08

For bipolar scales, the anchors for 1 and 5 are given in this sequence and separated by an ellipsis. E^2^–Epsilon-squared effect size estimate (Tomczak and Tomczak, 2014) with significance level of the Kruskal–Wallis test (^*^p < 0.05, ^**^p < 0.01, ^***^p < 0.001). ρ_ED_–Spearman correlation with ED based on the sum score criterion (Loevinger, 1998). Class means that do not share the same letter in the subscript are significantly different according to the Conover-Iman *post-hoc* test with Benjamini-Hochberg correction. Group labels: 1—“Indifference,” 2—“Ambivalence,” 3—“Acceptance,” and 4—“Seeking”.

The results of latent profile analyses revealed four latent profiles, and we created four participant groups based on their most likely class membership. Scores on all but three items tapping into lay theories of meaning (2, 13, and 15) differed significantly across the groups. The strongest differences were observed for items 5 (“Meaning is… An illusion, as life is absurd”), 6 (“Meaning is… A question useless to ponder on”), 16 (“The question of meaning… is a reasonable/pointless question”), 17 (“…has a lot to do/nothing to do with me”), and 19 (“…can/cannot be answered”) reflecting the personal salience of meaning. The mean scores reflect a more optimistic picture of views regarding life meaning in groups 3 and 4 and a more negative picture in the other two groups.

Participants in groups 1 and 2 tend to hold more negative views of meaning. They are less likely to understand meaning as a goal or a general direction of life (items 3, 4) and are more likely to think of it as an illusion (item 5) or as a question that is useless to ponder, because finding an answer is impossible (items 6, 19). They tend to view life meaning as something that is peculiar to a select few individuals (items 8 and 15), that can exist regardless of human choices (item 14), and is not necessary for life (item 9). The question of meaning appears to them as something they personally are not concerned with (items 17 “has nothing to do with me” and 18 “does not bother me at present”).

However, there are also important differences between these two groups. Participants in group 1 hold more extreme negative views dismissing the importance of meaning (items 16 “a pointless question” and 18 “does not bother me at present”). They also tend to see it as a given that does not depend on us and that might be common and universal for everyone (items 10 “is a given that does not depend on one” and 11 “one can discover or understand it”). These individuals distance themselves from meaning and, following Schnell (2010), we labeled this group “Indifference to Meaning.” Participants in group 2, however, are less likely to dismiss the question of meaning as a pointless one or one that does not bother them (items 16 and 18). They believe that meaning can only be felt or experienced, rather than rationally understood (item 7), but that it has to be created, rather than found (item 11). Based on this combination of a personal take on meaning and uncertainty regarding its existence, importance, and nature, we labeled group 2 “Ambivalence about Meaning.”

Participants in groups 3 and 4 tend to view meaning as a goal or a direction of human life (items 3, 4) that is real, rather than illusory (item 5). For them, meaning is necessary for one's life (item 9), it can be understood, rather than only felt (item 7), and this understanding is available to every person (item 8). The question of meaning is a reasonable and personally relevant one (items 16–19).

There are, again, some notable differences between these two groups. Participants in group 3 are somewhat less optimistic about the possibility of finding an answer to the question of meaning (item 19) and are more likely to consider the possibility of it being pointless (items 6 and 16). They are also somewhat less certain that meaning is relevant to their life (item 17) and dependent on their actions; instead, they are more likely to see it as a given that does not depend on one (item 10), that is common and universal (item 12), and that exists regardless of one's choices (item 14). For them, meaning appears to be real, but not necessarily vitally important or requiring any action. We labeled group 3 “Acceptance of Meaning.” Finally, participants in group 4 are more likely to see meaning as a conscious notion (item 1). They experience the question of meaning as a reasonable and useful question (items 6, 16) that can be answered (item 19) and that is directly related to their own lives (item 17). They see meaning as an individually-specific reality (item 12), a possibility that can be accomplished by the person, rather than a given (item 10), and as something that emerges as a result of one's life choices (item 14). Based on this existential view of meaning, we labeled group 4 “Seeking Meaning.”

There were no significant differences in education or age across the groups. However, gender distribution was not uniform [χ^2^ (3) = 19.43, *p* < 0.001, Cramer's *V* = 0.29]: there was a higher prevalence of female participants in group 3 “Acceptance of Meaning” (77.3%) and a higher prevalence of male participants in polar groups 1 “Indifference to Meaning” and 4 “Seeking Meaning” where female participants only comprised 51.2 and 52.8%, respectively. The gender distribution in group 3 “Ambivalence about Meaning” (61.2% of female participants) was close to the sample average.

The distribution of ED levels in the four groups with different lay theories of meaning did not significantly differ from uniform, based on the chi-square test [χ^2^ (12) = 18.64, *p* = 0.098, Cramer's *V* = 0.13]. However, it did not appear to be random (see Table 4): the prevalence of E2–E3 and E4 decreased and that of E5, E6, and E7–E9 increased in a perfectly monotonous manner from group 1 to group 4, in line with the increasing salience of meaning.

**Table 4:** Prevalence of individuals at different ED stages in each latent class.

**ED level**	**Prevalence of ED level in lay theory of meaning group**, ***N*** **(% of group)**				
	**1 “Indifference” (*****N*** = **41)**	**2 “Ambivalence” (*****N*** = **84)**	**3 “Acceptance” (*****N*** = **132)**	**4 “Seeking” (*****N*** = **105)**	**Sample average, %**
E2–E3	6 (14.6%)	8 (9.5%)	10 (7.6%)	4 (3.8%)	7.7%
E4	12 (29.3%)	16 (19.1%)	20 (15.2%)	9 (8.9%)	15.7%
E5	12 (29.3%)	31 (36.9%)	51 (38.6%)	44 (41.9%)	38.1%
E6	8 (19.5%)	22 (26.2%)	35 (26.5%)	33 (31.43%)	27.1%
E7-E9	3 (7.3%)	7 (8.3%)	16 (12.1%)	15 (14.3%)	11.3%

The ED stages were determined based on the sum rule (Loevinger, 1998).

A comparison of the groups on the other psychological variables is given in Table 5. The differences across the groups were significant for all variables, except for hedonic motives. The ED level based on the Total Protocol Rating was lowest in the “Indifference” group and highest in the “Acceptance” and “Seeking” groups. Predictably, the “Indifference” and “Ambivalence” groups had lower scores on the presence of meaning and search for meaning scales, as well as social wellbeing and overall wellbeing (interestingly, participants in the “Indifference” scored lowest on the search for meaning and highest on hedonic motives, whereas those in the “Ambivalence” group scored lowest on the presence of meaning, wellbeing, and eudaimonic motives; however, the pairwise differences between these groups suggesting potentially different patterns of a meaning crisis were not statistically significant). In addition to higher ED, individuals in the “Acceptance” and “Seeking” groups reported a higher presence of meaning and search for meaning, as well as higher wellbeing. Again, the picture was somewhat more positive in the “Seeking” group, but none of the pairwise differences between the two groups with a positive approach to meaning was significant.

**Table 5:** Cluster means and one-way ANOVA results for the study measures.

	**1 “Indiff.” (*N* = 41)**	**2 “Ambiv.” (*N* = 85)**	**3 “Accept.” (*N* = 132)**	**4 “Seeking” (*N* = 106)**	***F*(3)**	**η^2^**	**ρ**
1. Ego development (TPR)	4.68_a_	4.86_ab_	4.96_b_	5.02_b_	4.52^**^	0.036	0.18^**^
2. Hedonic motives	5.36_a_	5.08_ab_	4.99_ab_	4.90_b_	2.42^x^	0.021	−0.12^*^
3. Eudaimonic motives	5.57_ab_	5.21_a_	5.50_ab_	5.81_b_	7.37^***^	0.062	0.24^***^
4. Presence of meaning	4.07_ab_	3.69_a_	4.47_bc_	4.96_c_	12.07^***^	0.093	0.28^***^
5. Search for meaning	3.51_a_	3.98_ab_	4.52_bc_	4.64_c_	9.33^***^	0.073	0.24^***^
6. Emotional wellbeing	3.39_a_	3.49_a_	3.92_a_	3.88_a_	3.53^*^	0.029	0.15^**^
7. Social wellbeing	2.75_ab_	2.58_a_	3.03_b_	3.13_b_	5.70^**^	0.046	0.19^***^
8. Psychological wellbeing	3.51_ab_	3.30_a_	3.65_ab_	3.92_b_	5.74^**^	0.046	0.20^***^
9. MHC total score	3.21_ab_	3.08_a_	3.49_bc_	3.63_c_	6.14^***^	0.049	0.21^***^

^*^p < 0.05.

^**^p < 0.01.

^***^p < 0.001.

^x^*p* = 0.066.

Means that do not share a subscript letter are significantly different based on Tukey HSD *post-hoc* test (*p* < 0.05). The df for error is 358 for MHC and ED, 353 for MLQ, and 346 for HEMA. ρ–Spearman correlation between the group number (i.e., its rank in terms of concern for meaning) and the variable score.

To quantify the non-linear monotonous increase of scores in groups in line with the increasing salience of meaning, we also calculated Spearman correlations between the profile number and the self-report measure scores (see Table 5). The results indicate that the progression from group 1 to group 4 is associated with an increase in the presence of meaning, search for meaning, eudaimonic motives, ego development, wellbeing, and a decrease in hedonic motives.

## Discussion

The theories in the field of eudaimonia and ED aim to describe and explain overlapping phenomena of positive functioning that characterize a mature personality. However, until recently, the evidence of the empirical links between the models in the two fields was limited. Our study built on earlier work by Bauer and McAdams (2010) and Bauer et al. (2011) that revealed weak positive associations of ED with current wellbeing and extended it by confirming these associations using measures of eudaimonic wellbeing. Unfortunately, the modest sample size and the small number of individuals (*N* = 7) at the most advanced (E8–E9) ED stages did not allow us to fully replicate the findings of Bauer et al. (2011) who suggested that higher levels of wellbeing might only be observed at these stages. However, we observed a modest, yet significant difference in wellbeing scores between individuals at post-conventional stages (E7–E9) and those at earlier stages (E2–E6) (*d* = 0.33, *p* = 0.046).

In terms of effect size, the association of ED and wellbeing we found (*r* = 0.11–0.12) is in line with past studies, where the correlations of ED and psychosocial maturity with wellbeing have typically ranged from *r* = 0.00 to *r* = 0.22 (Bauer and McAdams, 2010; Bauger et al., 2021). The Eudaimonic Activity Model (Sheldon, 2018; Martela and Sheldon, 2019) proposes that subjective wellbeing can serve as a universal criterion of a life going well that reflects whether people's psychological needs are satisfied by rewarding experiences arising from eudaimonic activities. We side with that idea with one reservation: the activities needed to experience the same subjective state of basic needs satisfaction and wellbeing may qualitatively differ across individuals and within-person, across ages, as a function of the level of cognitive complexity and personality maturity that is captured by the ED model. SWB and eudaimonic subjective experiences, such as meaningfulness, provide subjective evaluations of whether the direction one is currently following in life is in line with one's deeper personal priorities and aspirations, but hardly allow one to evaluate how far one has progressed along the path of self-actualization, wherein one's needs, priorities, and aspirations continuously evolve (Maslow, 1971). In this sense, measures of psychosocial maturity and wellbeing appear to be complementary indicators reflecting related yet different aspects of life going well and, therefore, they are not supposed to correlate strongly.

Both of our hypotheses concerning the presence of meaning and eudaimonic motives as mediators of the relationship between ED and wellbeing were supported, indicating that all the variance shared by ED and wellbeing is explained by trait-level indicators of eudaimonic orientations. The positive association of ED with eudaimonic motives is in line with the idea that ED and growth orientation accompany each other (Bauer, 2011). Our data suggest that the increase in eudaimonic motives might be related to the transition from the Self-Aware (E5) to the Conscientious (E6) stage (*d* = 0.45, *p* < 0.001). The causal links between these processes are far from clear: the only longitudinal study to date (Bauer and McAdams, 2010) has only tested one direction of causality, showing that intellectual growth motives predict later ED. Unfortunately, the present study is limited by its cross-sectional design, but we hope that our findings may encourage future integration between these research areas.

The associations of ED with the presence of meaning and the search for it are also noteworthy. First, although the presence of meaning and the search for meaning are negatively correlated, ED is positively associated with both, reminding of Frankl's (1969) idea that existential meaning is different in every life situation and meaningful life involves actively searching for it every day. The latent profile analysis results corroborate these findings by showing that the two groups with a positive view of meaning in life, as well as higher ED and wellbeing scores, are characterized by a combination of the presence of meaning and the search for meaning. Only the presence of meaning, however, has emerged as a significant mediator of the ED—wellbeing relationship, suggesting that it is not the process of searching for meaning, but its outcome—a vision of one's life goal and priorities—that may bring the wellbeing benefits of eudaimonic growth. The choice between the parallel and the serial mediation models, which emerge as equivalent interpretations of our cross-sectional data, depends on whether one places the presence of meaning within the domain of eudaimonic orientations (on a par with eudaimonic motives) or within the domain of eudaimonic experiences (in terms of Huta, 2016) expected to emerge as outcomes of eudaimonic orientations. We believe that the MLQ operationalisation of this construct taps into both and that more recent, theoretically refined measures, such as the 3DM (Martela and Steger, 2022), could help to model meaning more discriminately.

Regarding the ED theory, the results offer new empirical evidence to specify the idea of meaning production, which is one of the foundations of growth that happens as the ED level increases (Loevinger, 1976). Our data reveal noticeable changes in the processes and outcomes of meaning production at higher ED levels: rather than being an abstract question or a universal given, meaning becomes a tangible and a personally experienced possibility constituting an integral part of one's daily life. Past studies have revealed that individuals at post-conventional ED stages exhibit higher levels of self-reflection (Pfaffenberger et al., 2011; Kostenko and Leontiev, 2018) and report more positive experiences of solitude (i.e., viewing solitude as a resource for self-knowledge and self-development—Ishanov et al., 2018) that could provide conditions for meaning-making.

The profiles we have identified contribute to this line of research, providing more insight into the inner dynamics of personality development. Increasing concern for meaning, viewing it as a reality, rather than an illusion, and a possibility, rather than a given, was associated with ED and wellbeing. The findings suggest that the presence of meaning and the search for meaning, despite being negatively correlated in the total sample, are both prerequisites for eudaimonic life that combines growth and wellbeing: indeed, as Frankl (1969) suggested, meaning cannot be found once and for all, one has to search for it anew in every life situation. The finding that hedonic motives mostly fail to exhibit negative associations with ED and meaning-related variables is consistent with the findings showing that pleasant life and meaningful life are relatively independent, rather than mutually exclusive pathways to wellbeing (Huta and Ryan, 2010; Schueller and Seligman, 2010).

Naturally, the present research has numerous limitations. First of all, the cross-sectional design does not allow making inferences concerning the temporal sequence and causality of changes in ED and eudaimonic functioning. Second, larger samples are needed to make reliable conclusions about individuals at post-conventional stages. Third, the changes in the patterns of meaning-making may not be the same across cultures. Fourth, MLQ and HEMA are limited in their validity, given the rich and multifaceted nature of meaning and eudaimonia as psychological constructs (Leontiev, 2013; Huta and Waterman, 2014; Martela and Steger, 2016). Future studies using more rigorous designs and sampling strategies, comprehensive measures, and more diverse cultural settings are needed to replicate and generalize these findings.

Nevertheless, we believe that the results show that the notion of eudaimonic functioning may explain the elusive associations between wellbeing and personality development. Existing work on ED suggests that self-report questionnaires may fail to capture the growing complexity associated with this process. The findings revealing the independent mediating effects of meaning and eudaimonic motives reveal perspectives for future studies that could shed more light on the mysterious process of attaining psychosocial maturity.

## Data availability statement

The datasets presented in this study can be found in online repositories. The names of the repository/repositories and accession number(s) can be found at: https://osf.io/pja8d/?view_only=7cd4719c4f144b77a4a5b92e53d970c4.

## Ethics statement

The studies involving human participants were reviewed and approved by HSE University Psychology Department Research Ethics Committee. Written informed consent for participation was not required for this study in accordance with the national legislation and the institutional requirements.

## Author contributions

EO and EV contributed to conception and design of the study and performed the statistical analyses. EV collected the data. VK and EV coded the WUSCT protocols. EO wrote the first draft of the manuscript. All authors contributed to manuscript revision, read, and approved the submitted version.
