# Supplementary material for: A growing concern for meaning: Exploring the links between ego development and eudaimonia
Source: Front Psychol. 2023 Mar 22;14:958721. doi: 10.3389/fpsyg.2023.958721 (PMC10075199; doi:10.3389/fpsyg.2023.958721)
Supplement: Supplementary file 1 [file Table_1.DOCX]

**Supporting Information**

Table SI.1. Fit of the measurement models (WLSMV estimator)

| Model | χ^2^ (df) | CFI | RMSEA [90 % CI] | SRMR |
| --- | --- | --- | --- | --- |
| WUSCT (CFA) | 242.62 (135) | .970 | .047 [.037; .056] | .038 |
| HEMA (CFA) | 357.82 (43) | .819 | .143 [.129; .156] | .068 |
| HEMA (ESEM) | 141.87 (34) | .938 | .094 [.078; .110] | .038 |
| MLQ (CFA) | 355.95 (34) | .950 | .162 [.147; .177] | .064 |
| MLQ (ESEM) | 299.11 (26) | .958 | .171 [.153; .188] | .028 |
| MHC (3-factor CFA) | 287.72 (74) | .949 | .089 [.079; .100] | .044 |
| MHC (3-factor ESEM) | 162.79 (52) | .974 | .077 [.064; .090] | .030 |

Table SI.2. Model fit statistics of the latent class models

| N classes | Npar | LL | Entropy | AIC | BIC | SABIC | VLMRT | BLRT |
| --- | --- | --- | --- | --- | --- | --- | --- | --- |
| 1 | 80 | -10130.40 | n/a | 20420.80 | 20732.57 | 20478.77 | n/a | n/a |
| 2 | 161 | -9723.69 | .865 | 19769.40 | 20396.84 | 19886.06 | p < .001 | p < .001 |
| 3 | 242 | -9504.00 | .894 | 19491.99 | 20435.11 | 19667.34 | p = .48 | p < .001 |
| 4 | 323 | -9366.73 | .915 | 19379.46 | 20638.25 | 19613.50 | p = .79 | p < .001 |
| 5 | 404 | -9241.08 | .940 | 19290.14 | 20864.60 | 19582.88 | p = .77 | p < .001 |
